# Supplementary figures and images for: Development and application of a CRISPR/Cas12a-based reverse transcription–recombinase polymerase amplification assay with lateral flow dipstick and fluorescence detection for Getah virus
Source: PeerJ. 2025 Oct 2;13:e20119. doi: 10.7717/peerj.20119 (PMC12497397; doi:10.7717/peerj.20119)

# Multicomponent Plot

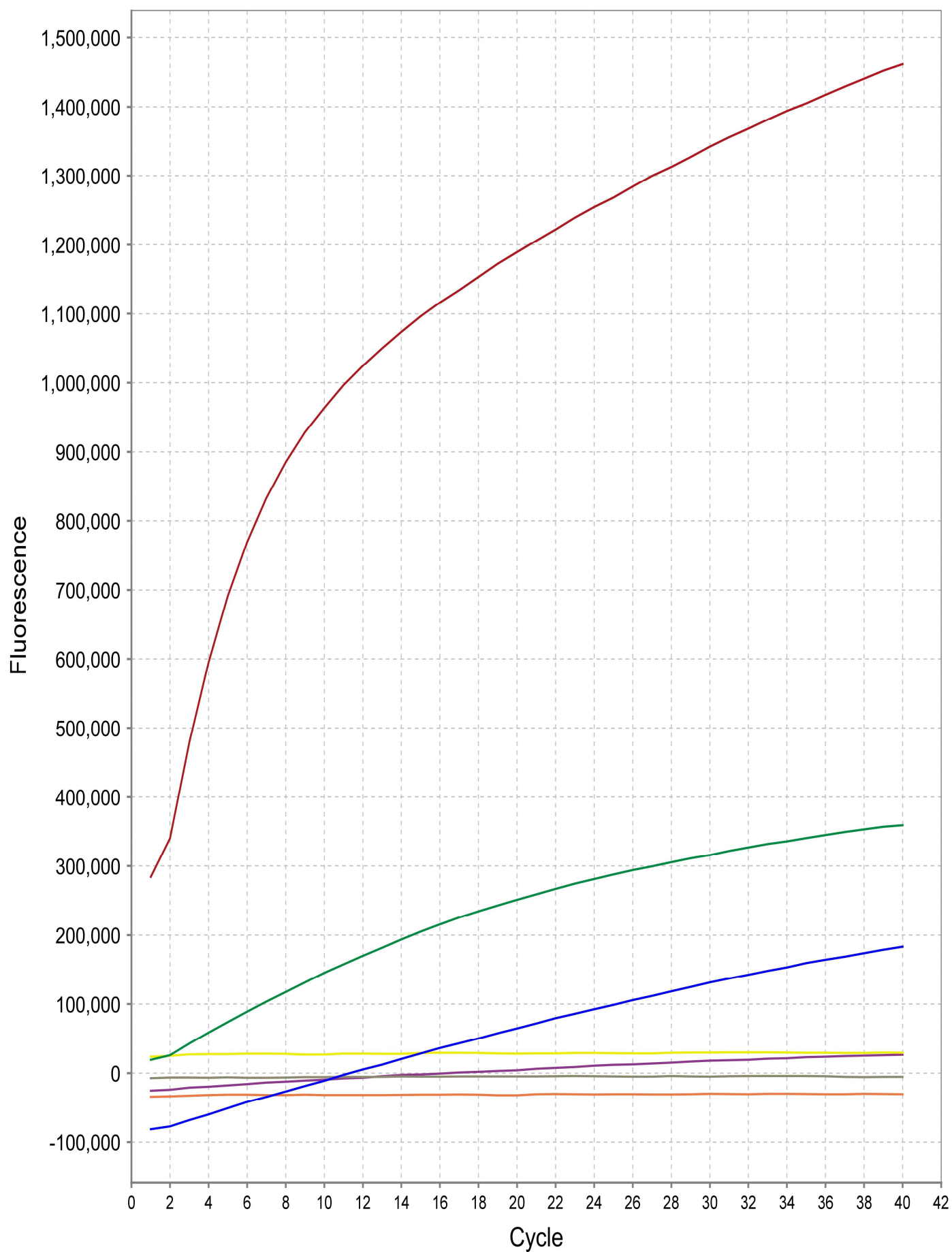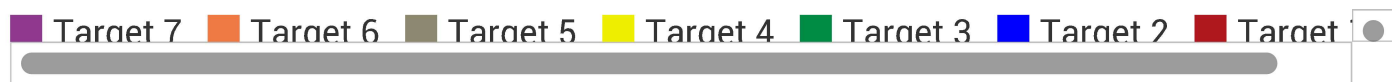

Supplement: Supplemental Information 2 [file peerj-13-20119-s002.pdf]

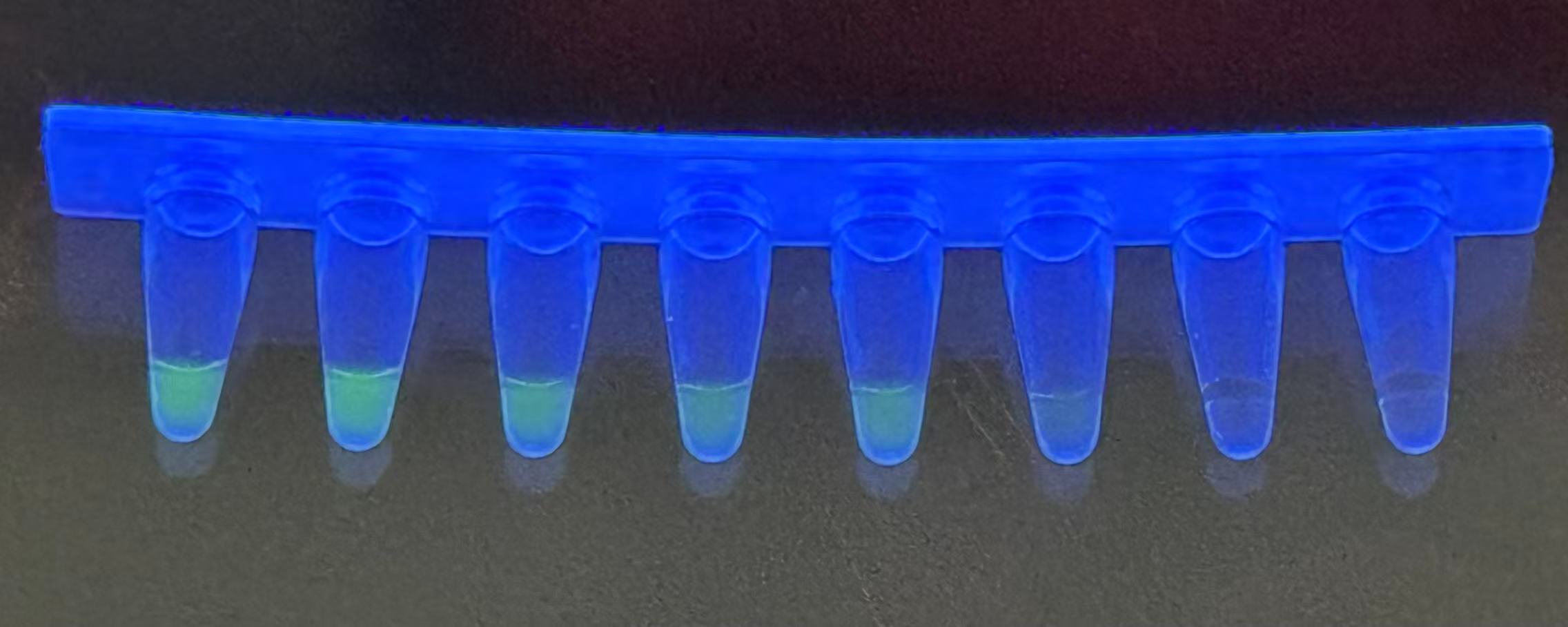

Supplement: Supplemental Information 4 [file peerj-13-20119-s004.jpg]

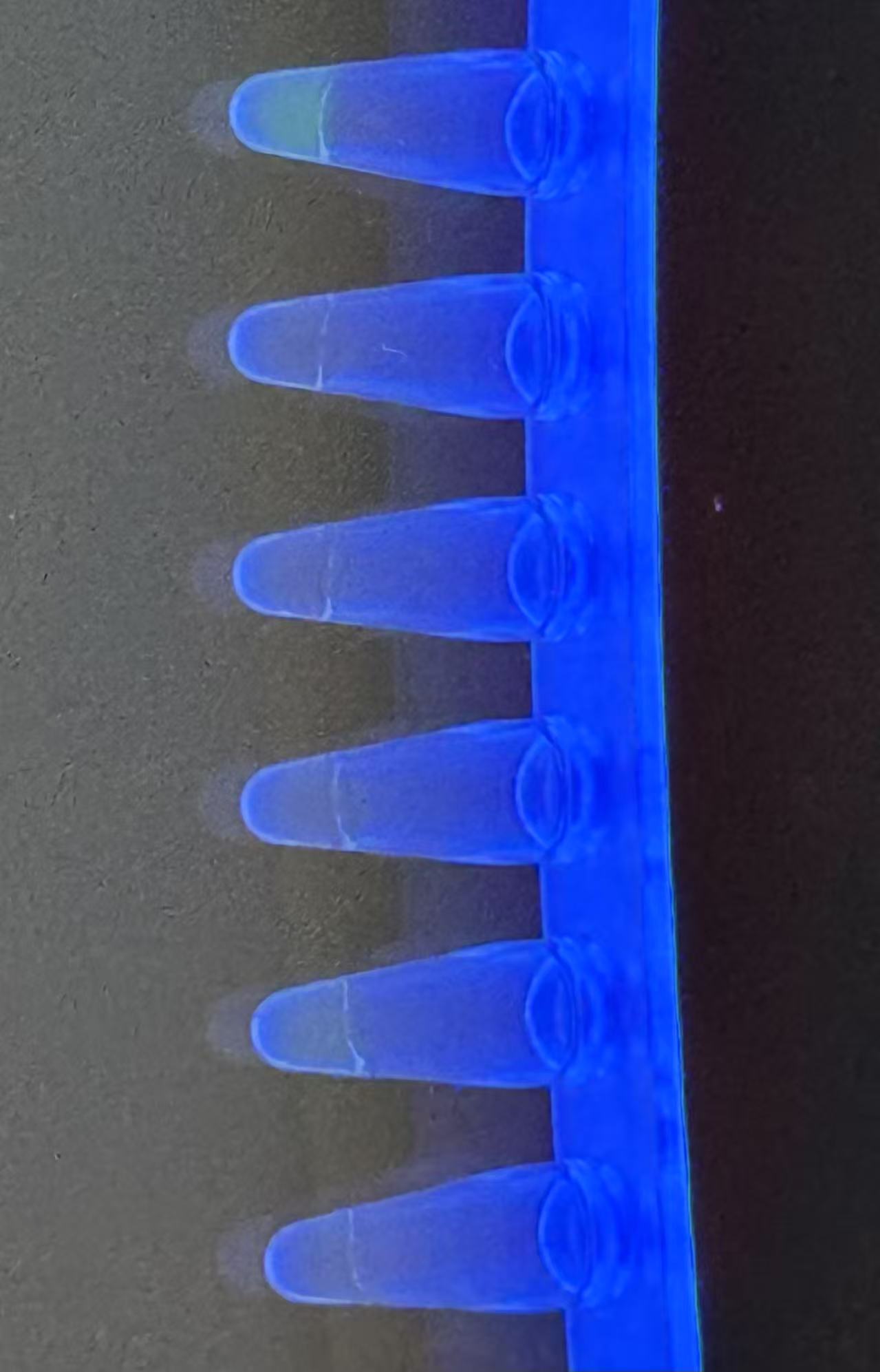

Supplement: Supplemental Information 6 [file peerj-13-20119-s006.jpg]
