# Supplementary material for: Development and application of a CRISPR/Cas12a-based reverse transcription–recombinase polymerase amplification assay with lateral flow dipstick and fluorescence detection for Getah virus
Source: PeerJ. 2025 Oct 2;13:e20119. doi: 10.7717/peerj.20119 (PMC12497397; doi:10.7717/peerj.20119)
Supplement: Supplemental Information 9 [file peerj-13-20119-s009.pdf]

Figure 2S:Repeatable experiment of RT-RPA-CRISPR/Cas12a-LFD specificity.C:control line, T:test line, NC:negative control

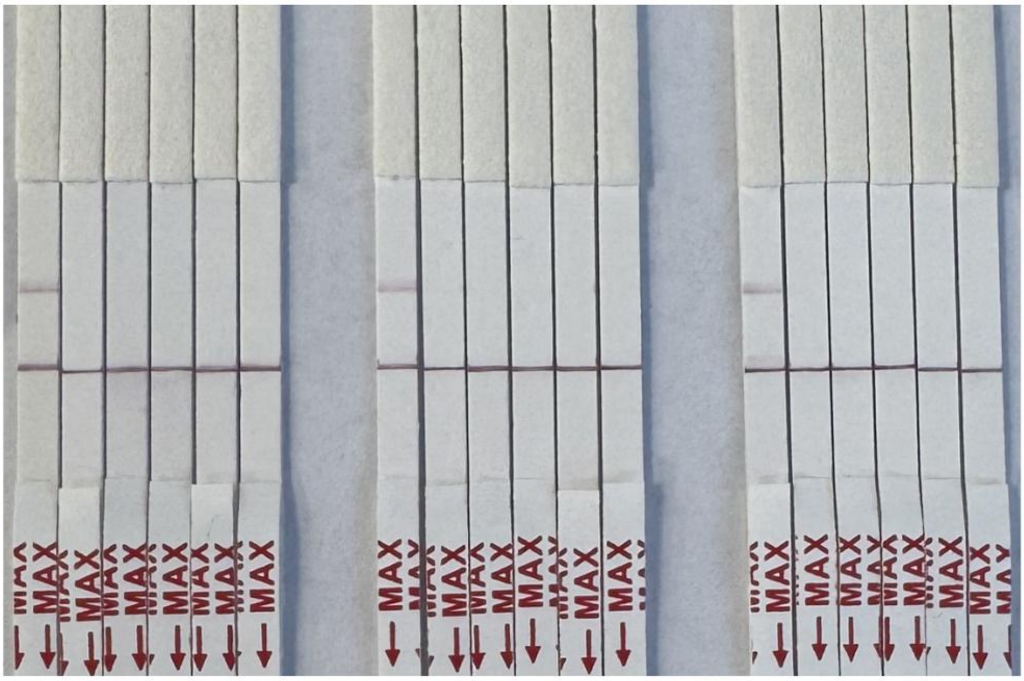

(1:GETV 2:JEV 3:PRV 4:PCV 5:CSFV 6:NC)
